# Minipool Caprylic Acid Fractionation of Plasma Using Disposable Equipment: A Practical Method to Enhance Immunoglobulin Supply in Developing Countries

**DOI:** 10.1371/journal.pntd.0003501

**Published:** 2015-02-26

**Authors:** Magdy El-Ekiaby, Mariángela Vargas, Makram Sayed, George Gorgy, Hadi Goubran, Mirjana Radosevic, Thierry Burnouf

**Affiliations:** 1 Shabrawishi Blood Bank, Shabrawishi Hospital, Cairo, Egypt; 2 Instituto Clodomiro Picado, Facultad de Microbiología, Universidad de Costa Rica, San José, Costa Rica; 3 Environmental and Food Pollutant Laboratory, Fayoum University, Fayoum, Egypt; 4 Saskatoon Cancer Center, College of Medicine, University of Saskatchewan, Saskatoon, Canada; 5 Human Protein Process Sciences, Lille, France; 6 Graduate Institute of Biomedical Materials and Tissue Engineering, College of Oral Medicine, Taipei Medical University, Taipei, Taiwan; University of California San Diego School of Medicine, UNITED STATES

## Abstract

**Background:**

Immunoglobulin G (IgG) is an essential plasma-derived medicine that is lacking in developing countries. IgG shortages leave immunodeficient patients without treatment, exposing them to devastating recurrent infections from local pathogens. A simple and practical method for producing IgG from normal or convalescent plasma collected in developing countries is needed to provide better, faster access to IgG for patients in need.

**Methodology/Principal Findings:**

IgG was purified from 10 consecutive minipools of 20 plasma donations collected in Egypt using single-use equipment. Plasma donations in their collection bags were subjected to 5%-pH5.5 caprylic acid treatment for 90 min at 31°C, and centrifuged to remove the precipitate. Supernatants were pooled, then dialyzed and concentrated using a commercial disposable hemodialyzer. The final preparation was filtered online by gravity, aseptically dispensed into storage transfusion bags, and frozen at <-20°C. The resulting preparation had a mean protein content of 60.5 g/L, 90.2% immunoglobulins, including 83.2% IgG, 12.4% IgA, and 4.4% IgM, and residual albumin. There was fourfold to sixfold enrichment of anti-hepatitis B and anti-rubella antibodies. Analyses of aggregates (<3%), prekallicrein (5-7 IU/mL), plasmin (26.3 mU/mL), thrombin (2.5 mU/mL), thrombin-like activity (0.011 U/g), thrombin generation capacity (< 223 nM), and Factor XI (<0.01 U/mL) activity, Factor XI/XIa antigen (2.4 ng/g) endotoxin (<0.5 EU/mL), and general safety test in rats showed the in vitro safety profile. Viral validation revealed >5 logs reduction of HIV, BVDV, and PRV infectivity in less than 15 min of caprylic acid treatment.

**Conclusions/Significance:**

90% pure, virally-inactivated immunoglobulins can be prepared from plasma minipools using simple disposable equipment and bag systems. This easy-to-implement process could be used to produce immunoglobulins from local plasma in developing countries to treat immunodeficient patients. It is also relevant for preparing hyperimmune IgG from convalescent plasma during infectious outbreaks such as the current Ebola virus episode.

## Author Summary

Plasma-derived immunoglobulin G (IgG) is on WHO’s Essential Medicines List, yet developing countries face severe shortages of this critical treatment. Infusion of IgG prepared from locally-collected plasma provides an advantageous mix of antibodies to viral and bacterial pathogens found in the living environment, and this can reduce recurrent infections in immune-deficient patients. We developed a simple manufacturing process using disposable equipment (blood bags, hemodialyzer, and filters) to isolate immunoglobulins from minipools of 20 plasma donations. This process yields a ca. 90% pure virally-inactivated immunoglobulin fraction at 50–60% recovery. Anti-hepatitis B and anti-rubella immunoglobulins were enriched fourfold to sixfold. The product was free of *in-vitro* thrombogenic and proteolytic activity, confirming its expected clinical safety profile. Virus validations showed caprylic acid treatment robustly inactivated or removed infectivity of lipid-enveloped viruses, including human immunodeficiency virus (HIV) and hepatitis C virus model. This simple and cost-effective process is implemented in Egypt to prepare experimental batches for clinical evaluation. It can enhance immunoglobulin supplies to treat immunodeficient patients through passive transmission of antibodies directed against local pathogens. The method requires minimal training and reasonable infrastructure, and is a practical means to prepare convalescent hyperimmune IgG during infectious outbreaks such as the current Ebola episode.

## Introduction

Plasma products to treat congenital bleeding and immunological diseases are made in industrialized countries using complex technologies unavailable in the developing world [1]. Low- to medium-income countries may have little or no access to these life-saving products; these nations urgently need practical processing methods to produce them affordably. We have introduced the concept of small-scale (“minipool”) plasma processing methods implementable with minimum infrastructural requirements. We developed viral inactivation and protein purification technologies in single-use equipment to prepare virally safe solvent/detergent-filtered (S/D-F) plasma for transfusion as well as minipool S/D-F cryoprecipitate to treat bleeding disorders [2–4]. Similarly simple technologies are desperately needed to make safe immunoglobulin G (IgG), a product on the Essential Medicine List of the World Health Organization, to treat immune-deficient patients. Thus we describe here a small-scale caprylic acid IgG fractionation process that requires minimal capital investment and uses disposable equipment. This production approach could increase the supply of IgG in developing countries and improve treatment of immunodeficient patients. It is also a realistic approach to consider in the preparation of convalescent immunoglobulins during infectious outbreaks such as the current Ebola virus epidemic [5,6].

## Methods

### Plasma Preparation

Whole blood was collected with CPD-A anticoagulant/preservative solution (ratio: 14ml/100ml of blood) from regular volunteer non-remunerated donors at Shabrawishi Hospital Blood Bank (Giza, Cairo, Egypt). Donors received information prior to donation in compliance with national regulations. The procedure was approved by the Institutional Review Board from Cairo University (Number N-5–2014). The blood bank is licensed (license number N°7) by the General Directorate for Blood Transfusion Affairs, Ministry of health and is ISO certified (ASR number 1230).

Non-leuco-reduced blood was centrifuged at 3600x *g* for 12 minutes within 4 hours of collection. Plasma was transferred into storage bags, frozen in a -40°C freezing room, and stored at ≤-25°C for a maximum of 12 months.

### Minipool IgG Fractionation

**Fig 1:**
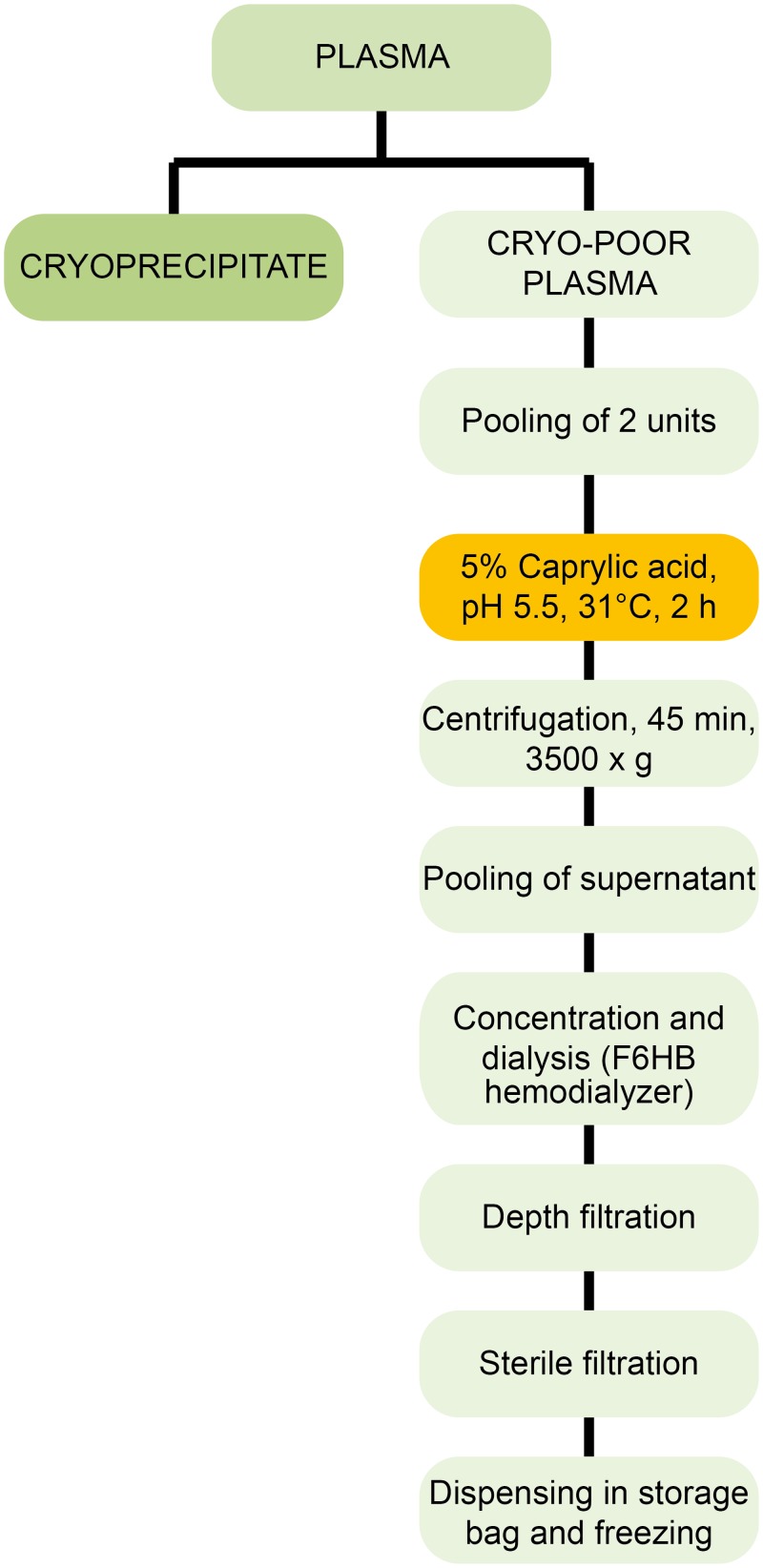
Preparation scheme of the IgG-enriched plasma fraction.

The preparation of the IgG fraction is summarized in Fig. 1. Plasma from 20 blood donations tested for anti-A and anti-B titer < 32 (Micro Typing Cards with NaCl; DiaMed AG, Cressier sur Morat, Suisse), or from the same blood group, were subjected to in-bag cryoprecipitation [2,7]. The cryoprecipitate-poor supernatant (approximately 200mL) was transferred into a transfusion bag, frozen and stored at <-30°C. Supernatants were thawed at 30–35°C. After thawing, caprylic acid (Merck, Darmstadt, Germany) was added within one minute to each bag under constant manual stirring to 5% (v/v) final concentration, pH 5.5 +/- 0.1, and the mixture incubated at 31+/- 0.5°C for 90 minutes at 150 rpm in a temperature-controlled shaker-incubator (Lab Therm LT-W, Kühner, Switzerland) [2]. Precipitated proteins were removed by centrifugation (KR4i, Jouan, St Herblain, France) at 3500x *g* for 45 minutes. The clear supernatants (approximately 2.8 L) were pooled under laminar flow into a SD Virus Inactivation Bag Cascade (VIPS SA, Colombier, Switzerland) and concentrated (typically 60 g/L) using a sterile single-use hemodialyzer (F6HB, Fresenius, Bad Homburg, Germany), a hemodialysis pump and monitoring equipment (Terumo BCT, Lakewood, CO, United States). The solution was progressively diluted with 5 volumes of sterile pyrogen-free saline solution and subjected to diafiltration to remove caprylic acid and concentration. The Ig fraction was centrifuged (Jouan) at 3500x *g* for 45 minutes at 2–4°C to remove any particulates, then filtered by gravity through a pyrogen-free pharmaceutical-grade BC0025L60SP03A cartridge (3M Cuno, Cergy-Pontoise, France) and a 0.2 μm Mini-Kleenpak sterilizing filter (Pall Corporation, Dreieich, Germany) and directly dispensed under laminar flow into sterile plastic storage bags and stored frozen at <-25°C.

### Protein Characterization and Purity Profile

Total protein was determined by Biuret (Protein Kit 110307, Merck Millipore, MA, USA). Zone electrophoresis was performed on agarose gels (Hydragel 7 protein kit, Sebia, Evry, France), staining with amidoblack and densitometric analysis by a semi-automated Hydrasys instrument (Sebia). Sodium dodecylsulfate polyacrylamide gel electrophoresis (SDS-PAGE), under non-reducing and reducing conditions, used 4%~12% Bis-Tris Gel (NuPAGE, Novex Life Technologies, CA, USA) as before [8]. Albumin was measured photometrically using bromocresol green (DiaSys Diagnostic Systems, Holzheim, Germany). IgG, IgA, and IgM were determined by immunoturbidimetry [9]. Anti-hepatitis B and anti-rubella immunoglobulins G titres were determined using Architect Anti-HBs Reagent and Architect Rubella IgG reagent, respectively (Abbott Laboratories, North Chicago, IL, USA). Molecular size distribution was analyzed by size exclusion chromatography on a TSKGel G3000SWXL column (7.8mm ID X 30 cm L) protected with a TSKGel guard column (6.0mm ID X 4.0 cm L), equipped with isocratic pump model SDS 9414, UV-VIS detector model S3210, Rhiodyne manual injector and PeakSimple Chromatography Data System SRI Model333 as data integrator (Schemback, Germany). The mobile phase was 0.1 M sodium sulfate, 50 mM sodium acetate, 0.05% sodium azide, pH5, flow-rate was 0.5 ml/min, and the detection wavelength was 280nm. Thrombin generation assay (TGA) used Technothrombin fluorogenic substrate and RC High reagent (Technoclone, Vienna, Austria), and prekallikrein activator (PKA), plasmin, thrombin, thrombin-like amidolytic activities used S-2302, S-2251, S-2238, and S-2288 chromogenic protease substrates (Chromogenix, Milan, Italy), respectively [10]. Factor XI coagulant activity was measured by one-stage thromboplastin time coagulation assay with human factor XI—deficient and reference plasma (DiaMed, Cressier, Switzerland), and FXI/FXIa antigen with Human Factor XI quantitative sandwich ELISA (Abcam, Cambridge, UK) as before [10]. Endotoxins were determined by the LAL assay. A licensed human IgG preparation produced by a combined ethanol fractionation-chromatography process was used as a control. Data are expressed as the mean ± standard deviation.

### Caprylic Acid

The 0.5-mL samples were mixed with 1mL ice-cold methanol and incubated overnight in a deep freezer at -80°C. Samples were centrifuged at 4000x *g* for 20 minutes and 1 mL of the supernatant was taken and filtered through a 0.45μm syringe filter to a 1.5 ml clean tube. Samples were processed and analyzed by HPLC (Schemback SFD GmbH, Bad Honnef, Germany) equipped with analytical pump (SFD 9414), UV/VIS detector (S 3210), Rheodyne manual injector model 7725i, Peak Simple Data System model 333 (SRI, Torrance, California, USA), and Luna 5u, C8(2) 100Å (150 mm x 4.6 mm) column chromatography (Phenomenex, Torrance, USA); 0.1% trifluoroacetic acid (TFA) in a 80:20 mixture of methanol (Fisher Scientific, UK) and water was used as mobile phase at 0.8 mL/min. Caprylic acid detection was done at 214nm wavelength

### Di (2-ethylhexyl) Phthalate (DEHP) Assay

Di (2-ethylhexyl) phthalate (DEHP) was assessed on the starting plasma and final IgG. Samples were processed and analyzed as before [2] by HPLC (Schemback SFD GmbH, Bad Honnef, Germany) equipped with analytical pump (SFD 9414), UV/VIS detector (S 3210) at a wavelength of 202 nm, using a Lichrospher 100 RP 18–5μ (250 mm x 4.6 mm) column (CS-Chromatographie Service GmbH, Langerwehe, Germany). A mixture of 85:15 of acetonitrile and methanol (Merck) was used as mobile phase at 1.5 mL/min for 8 minutes analysis time.

### Viral Reduction Studies

The capacity of the caprylic acid treatment to inactivate/remove viruses was assessed at Texcell (Evry, France), a specialized laboratory working under GLP compliance awarded by the ANSM, France’s National Agency for Medicines and Health Products Safety. The process was scaled-down by a factor of 10 (40 mL). Validations were performed in duplicate under worst-case conditions using cryo-poor plasma as starting material, 4.8% caprylic acid, and a temperature of 28.5–30.5°C. The study followed Good Laboratory Practices and CPMP recommendations [11]. Plasma tested negative for HBsAg; HIV-1/HIV-2 Ab+P24 Combo assay; Anti-HCV by Abbott Architect Chemiluminescence (Abbott Laboratories); HBV, HIV and HCV individual-donor-Nucleic Acid Test (NAT) (Tigris; Grifols Diagnostic Solutions Inc., Emeryville, CA, USA) using the Procleix Ultrio assay. Cryo-poor plasma samples were prepared at the Shabrawishi Hospital Blood bank, frozen at -30°C and shipped with dry ice to Texcell. HIV-1 (Lai strain), bovine viral diarrhoea virus (BVDV; NADL strain; ATCC VR-534), and pseudorabies virus (PRV; Aujeszky disease virus; Kojnock strain; ATCC VR-135) were used for spiking, and P4-CCR5, MDBK cell lines (ATCC CCL-22) and Vero (Molecular Virology Laboratory, Institut Pasteur, Paris, France), respectively, for titration assays. Cryo-poor plasma was transferred into the reaction container. When temperature reached 28.5–30.5°C, the material was spiked with virus-inoculum (2.0% [v/v]). Spiked starting material was homogeneized and positive controls were collected; 4.8% (v/v; final concentration) caprylic acid was added in less than one minute. The spiked solution was kept at 28.5–30.5°C under continuous transversal agitation. Samples were taken right after caprylic acid addition (T0) and at 15, 45, 60, and 120 minutes after, and were immediately centrifuged at 3500x *g* for 45 minutes at 4°C. Supernatants were recovered and diluted 30 folds (BVDV) or 50 folds (PRV and HIV) with culture medium to stop the reaction, and were frozen and stored at -70°C. Control samples prepared under these conditions were verified not to induce cellular toxicity. Spiked samples were titrated by validated end-point dilution assay, and the viral clearance of the steps was assessed in terms of infectivity. Viral titers were calculated and expressed as 50% tissue culture infective dose per milliliter (TCID_50_/mL) using the Sperman Kaber formula. Infectious titers were calculated at the non-interfering dilution after large volume titration.

### General Safety Tests

The safety of the purified IgG was evaluated in Sprague—Dawley rats as described before [2], after approval from the National Cancer Institute (Cairo, Egypt), where the study was performed. It was conducted according to institution guidelines on animal studies and following recommendations specified in the Code of Federal Regulations Title 21, except that the injection was done intravenously, not intraperitonally, and the observation period was 14 days instead of 7. Twenty-one healthy rats weighing 80–100 g and not used previously for any test purpose were divided into three groups of seven rats. Animals received a dose of 6.5 mL/kg of saline, commercial IgG (control) or minipool IgG. The rats were observed once daily for abnormal behavior or clinical signs. Body weight, and consumption of water and food was recorded at 10 time-points over the observation period. The study was only observational and did not require anesthesia, sacrifice nor dissection.

## Results

**Fig 2:**
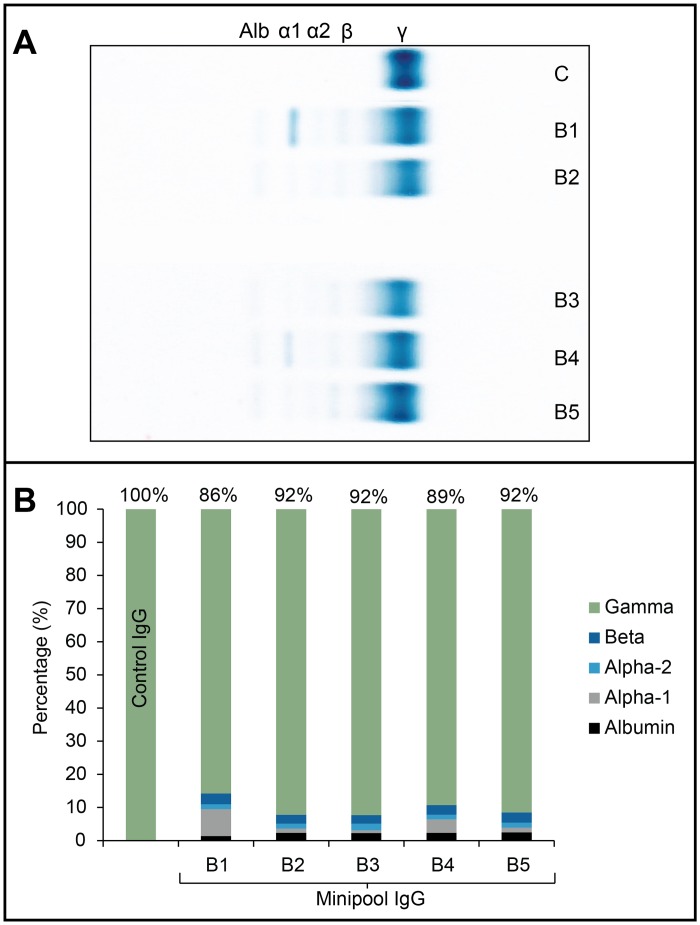
Zone electrophoresis. A: Patterns of 5 consecutive batches of minipool IgG-enriched plasma fractions (Minipool IgG batches B1 to B5) and control IgG (C) showing the separation between albumin (Alb) and alpha-1 (α-1), alpha-2 (α-2), bêta- (β) and gamma- (γ) proteins. B: densitographic analysis showing the percentage (%) of albumin, alpha-1, alpha-2, bêta, and gamma proteins in 5 batches (B1–B5) of minipool IgG. Control: control IgG.

**Fig 3:**
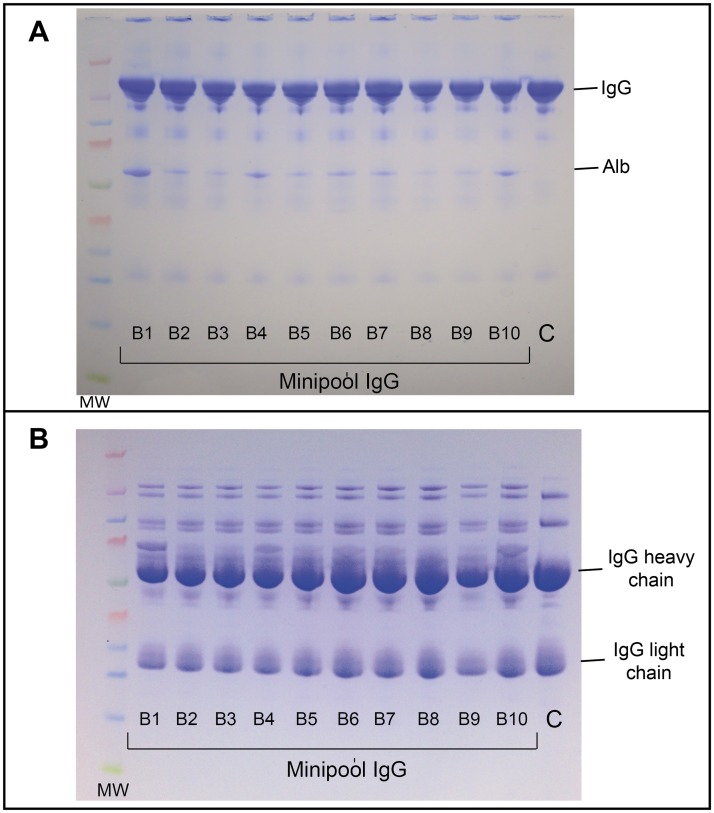
Sodium dodecyl sulfate-polyacrylamide gel electrophoresis. Patterns of 10 consecutive batches of minipool IgG-enriched plasma fractions (Minipool IgG batches B1 to B10) and control IgG (C) under non-reducing (A) or reducing (B) conditions. Control: control IgG; IgG: immunoglobulin G; Alb: albumin. MW: molecular weight markers (kDa).

The main characteristics of the final preparations are summarized in Table 1. The product was clear, with a slight bluish color, and not turbid. Sodium content and osmolality were close to physiological value. Mean protein concentration was 60.2 g/L, with a content of gamma-globulins close to 90%, and traces of albumin, alpha-1, alpha-2, and bêta-proteins revealed by zone electrophoresis (Fig. 2A and B). The relative abundance of IgG was 82–85%, IgA 11–13%, and IgM 4–5%, close to the physiological proportion. Albumin was less than 3 g/L. Content of high molecular weight proteins/aggregates was less than 3% and monomers and dimers more than 90% by HPLC. Anti-A and anti-B isoagglutinin titer in all batches was less than 1/32. Titer of anti-hepatitis B and anti-rubella immunoglobulin G showed enrichment factors compared to plasma of 5.8 and 4.1, respectively. Proteolytic and thrombogenic activity were also assessed. Mean PKA was 6.1 ± 1.1 IU/ml (control: 3 IU/ml), well below the maximum limit of 35 IU/ml in the European Pharmacopoeia. TGA data showed a peak thrombin of 0–223 nM (control: 56.8 nM) below the threshold value of 350nM associated with thromboembolic activity in some IVIG preparations. Mean plasmin was 26.3 mU/mL (control: 20.3 mU/mL), thrombin 2.5 mU/mL (control: 24 mU/mL), thrombin-like proteolytic activity 0.011 U/g protein (control: 0.06 U/g protein), Factor XI activity < 0.01 IU/mL, and Factor XI/XIa 2.4 ng/g protein. SDS-PAGE (Fig. 3) under non-reducing conditions (A) evidenced that most proteins migrated at a MW close to 150–160 kDa (immunoglobulins G and A). Minor protein bands were detected at MW close to 25, 60, and 80–90 kDa. Under reducing conditions (B), two major protein bands with MW of 50 and 25 kDa (immunoglobulin G heavy and light chains, respectively) were detectable. The minipool IgG pattern was similar to control apart for additional protein bands with MW of approximately 150–160, 90, and 70kDa under reducing conditions. Caprylic acid in the final preparation was <750ppm, and DEHP <5ppm. Endotoxin content was less than 0.5 EU/ml.

**Table 1:** Properties and specifications (or range) of the IgG-enriched plasma fraction.

Parameters	Results	Method
**Formulation**	Frozen	-
**Appearance**	Clear, bluish, no visible particles*	Visual observation
**pH**	5.4–5.6*	pH meter
**Na, mmol/L**	148.2 +/- 1.6*	Chemical analyzer
**K, mmol/L**	2.4 +/- 0.1*	Chemical analyzer
**Osmolality, mosm/kg**	295 +/- 15*	Osmometer
**Total proteins, g/L**	60.5 ± 16.7**	Biuret
**Gamma globulins, %**	90.2±2.8**	Zone electrophoresis
**Immunoglobulin repartition**		
**IgG, %**	83.2 ± 1.1**	Immunoturbidimetry
**IgA, %**	12.4 ± 1.0**	Immunoturbidimetry
**IgM, %**	4.4 ± 0.6**	Immunoturbidimetry
**Albumin, g/L**	< 3*	Bromocresol green
**Aggregates, %**	< 3*	HPLC
**Monomers and dimers, %**	> 90%*	HPLC
**Anti-A/Anti-B, titer**	< 1/32*	Micro-typing
**Anti-HBs, mIU/mL (enrichment factor)**	330 +/- 421 (5.8 +/- 2.7)*	Architect, Abbott
**Anti-rubella, mIU/ml (enrichment factor)**	322. 1 +/- 111.9 (4.1 +/- 1.3)*	Architect, Abbott
**PKA, IU/mL (3% protein concentration)**	6.1 ± 1.1**	S-2302**
**Thrombin generation, peak thrombin, nM**	<350 (0–223)**	Technothrombin assay, RC high reagent
**Plasmin, mU/mL**	26.3 ± 2.4**	S-2251
**Thrombin, mU/mL**	2.5 ± 2.4**	S-2238
**Thrombin-like activity, U/g**	0.011 ± 0.008**	S-2288
**Factor XIc IU/mL**	< 0.01*	Coagulation assay
**Factor XI/XIa Ag, ng/g protein**	2.4 ± 0.9**	Sandwich ELISA
**Caprylic acid, μg/mL**	< 750*	HPLC
**DEHP, ppm**	< 5*	HPLC
**Endotoxin, EU/ml**	< 0.5**	LAL test
**Sterility**	Pass*	Sterility test (growth medium)

* N = 10;

** N = 5 (consecutive batches)

Viral validation data (Table 2) showed that (a) viral infectivity was not affected after spiking to the starting material, and (b) HIV-1, BVDV, and PRV inactivation was fast and complete within 15 minutes after caprylic acid addition. Reduction factors (duplicate experiments) were > 5.69 and > 5.74 log for HIV-1, > 5.23 and > 5.35 log for BVDV, and > 5.10 and >5.10 log for PRV.

**Table 2:** Log removal factors of HIV-1, BVDV, and PRV during caprylic acid treatment (duplicate experiments).

Virus	HIV	BVDV	PRV
**Strain**	Lai	NADL	Kojnock
	Infectious titer in spiked starting material*		
**Run A**	2.35 x 10^6^	1.31 x 10^7^	2.88 x 10^6^
**Run B**	2.79 x 10^6^	1.73 x 10^7^	2.88 x 10^6^
	Infectious titer after caprylic acid treatment*		
**Run A**	< 4.86**	< 7.72 x 10^1^ **	< 2.28 x 10^1^ **
**Run B**	< 4.86**	< 7.72 x 10^1^ **	< 2.28 x 10^1^ **
	**Mean reduction factor (log 10)**		
**Run A**	>5.69	>5.23	>5.10
**Run B**	>5.74	>5.35	>5.10

*Viral titers are expressed as TCID_50_/mL.

**Infectious titers at the non-interfering dilution after large volume titration.

General safety tests did not induce rat mortality nor behavioral changes in the three groups and there was no significant difference in body weight increase rate over 7 days (Table 3). The water and food consumption was not noticeably different among the three groups, ranging between 4–13% and 9–14% of total available, respectively, over the 14 days of observation post infusion.

**Table 3:** Percent increase in body weight of rats (3 groups of 7 rats) treated with saline, commercial IgG, or minipool IgG.

Treatment	Control		Commercial IgG		Minipool IgG	
Period (day)	Mean	± SD	Mean	± SD	Mean	± SD
**1**	19.29	10.58	7.57	16.92	11.43	6.27
**2**	24.29	15.03	11.57	17.70	16.00	7.87
**5**	35.86	13.33	26.00	19.58	31.43	7.59
**6**	30.00	16.67	18.14	20.91	18.14	7.90
**7**	35.14	15.23	26.14	16.45	27.57	9.52
**8**	41.71	15.84	26.00	20.18	32.00	8.77
**9**	43.29	15.96	24.14	21.67	33.00	9.93
**12**	47.14	18.21	25.71	13.78	34.86	8.86
**13**	48.57	18.06	35.00	19.16	39.71	10.00
**14**	52.14	20.41	38.86	22.43	42.43	9.62

## Discussion

Polyvalent IgG, the leading plasma product [12], is manufactured from thousands of liters of US or European plasma pools fractionated in highly sophisticated facilities using complex and highly regulated large-scale technologies [1,13]. This IgG product is in short supply and has a mix of antibodies that is not adapted to the treatment of patients in tropical areas, who are exposed to different pathogens. It is therefore crucial to develop small-scale, easy-to-use technologies adapted to process the plasma available in non-Western countries. We show here that a minipool human IgG-enriched fraction can be prepared using a simple process run in disposable CE-marked equipment. The technique relies on caprylic (octanoic) acid precipitation of non-Ig molecules [14,15]. Caprylic acid is already used to prepare licensed IgG or IgM preparations from precipitates II+III [16,17], II [18], or III [18]; 5%-pH 5.5 caprylic acid precipitation is also used to produce horse plasma-derived therapeutic antivenom immunoglobulins [19,20], and this process may become an alternative to chromatography for monoclonal antibodies production [21].

The minipool Ig fraction contained approximately 90% Ig, with a ratio of IgG/IgA/IgM similar to plasma. The content in IgA does not constitute a risk for primary immunodeficient patients who cannot develop anti-IgA. The preparation should not be infused to IgA-deficient patients, although such risks were recently highlighted as being not evidence-based in many patients [22]. Residual proteins included albumin, alpha-1, alpha-2, and bêta-proteins. Aggregate content was low (below 3%) in compliance with requirements for commercial intravenous IgG. It is important to ensure that the anti-A /-B titers of the minipool IgG is consistently low, especially in a situation where the preparation would be used in hematologically challenged recipients, as is the case of Ebola patients. All batches prepared here had a titer below 1:32, less than the limit of 1:64 in the European Pharmacopeia. One means to reduce the ABO isoagglutinin titer is to mix plasma donations for each batch based on a ratio of 30% group A, 30% group B, 20% group AB and 20% group O, an approach already done for a universal pooled plasma for transfusion [23]. Another means we have now implemented is the preparation of minipool IgG from specific blood group plasma donations allowing transfusion to matched blood group patients. The preparation was free of proteolytic activity and had PKA levels within European Pharmacopoeia limits. Recent thromboembolic events associated with intravenous or sub-cutaneous IgG [24,25] prompted us to check most particularly for procoagulant markers [26] especially in situations when such minipool Ig preparation would be used in individuals (e.g. ebola patients) experiencing disseminated intravascular coagulation. Results using TGA, the current preferred assay for assessing the thrombogenicity of IgG [25,27,28], chromogenic substrates for thrombin and thrombin-like activity, and ELISA and coagulant assays to detect FXI and FXIa strongly suggested that the preparation is devoid of relevant *in vitro* thrombogenic and proteolytic activity. Previous spiking experiments using cryoprecipitate-poor plasma had shown that 5%-pH5.5 caprylic acid incubation inactivates/precipitates FXI/FXIa [9], confirming our findings. We could not assess Fc fragment integrity and anticomplementary activity, but the fact that immunoglobulins are not precipitated during the whole procedure argues in favor of unlikely molecular alteration and aggregation [12]. The disposable hemodialyzer was effective in removing caprylic acid to undetectable levels. It may also contribute to removal of DEHP plasticizer, together with the adsorption filter used prior to sterile filtration and dispensing, as previously found for a charcoal filter [2].

Viral safety of the preparation relies on proper donor screening, serological testing of donations [29,30] and, when feasible, single-donor multiplex NAT testing for HIV, HBV and HCV [31], as was done in this research. Most importantly, manufacturing processes should include one or two dedicated viral inactivation steps, a major tripod of viral safety [30,32]. Caprylic acid treatment is known to be a robust viral reduction treatment for both human [16,18,33] and horse-derived IgG [34,35]. Our study confirms that at the concentration and pH used in this work, this is highly effective against lipid-enveloped viruses, as > 4 log of HIV, BVDV and PRV were inactivated within 15 minutes of treatment. Implementing a pH4 incubation step is readily possible as a second inactivation step for lipid-enveloped viruses [30]. The small size of the pool (20 donations) and the neutralizing activity of potential antibodies against hepatitis E or A (HAV) viruses and parvovirus B19 reduce the risks from non-enveloped viruses. Although we did not perform such evaluation, some removal of non-enveloped viruses may occur during caprylic acid precipitation, as reported for parvovirus and HAV by others [16,17,36]. In addition, dedicated virus removal by 20–35nm nanofiltration, as well as duplex nucleic acid testing for HAV and Parvovirus B19 could be considered to improve the safety margin, especially if larger batches are produced, to make these additional steps more cost-effective [37]. Reproducible IgG recovery (55–65%, corresponding to about 4.5 to 5 g of IgG/L plasma) was achieved, consistent with recovery of antivenom immunoglobulins from horse plasma [19].

This process could be implemented readily in blood establishments or national service centers after appropriate operator training and basic equipment acquisition. The caprylic acid treatment is performed in a closed-bag system under continuous gentle transversal agitation in a thermostated shaker incubator. Laminar flow cabinets are used for additions of reagents to protect against bacterial contamination. Blood bank centrifuges are used for centrifugation. Concentration of IgG and caprylic acid removal are done using commercially available single-use pyrogen-free hemodialyzer. These devices are easy to use, do not present cleaning-related cross-contamination risks, and are affordable (about US$10 per unit). IgG concentration and dialysis fraction is fast (typically 120 minutes to concentrate 3.1 to 3.2 L of supernatant IgG, and 90 minutes to perform 5 dialysis cycles), and yields a clear solution free of particles. The bluish color is typical of processes using caprylic acid and is likely due to the presence of residual ceruloplasmin [38]. The resulting concentrate is clarified by simple gravity, without pumps, on a pyrogen-free single-use adsorptive filter connected online to a sterilizing 0.2μm filter, as is also done for S/D-F plasma and S/D-F cryoprecipitate [2]. The process can use whole plasma, cryoprecipitate-poor plasma or prothrombin complex-depleted cryoprecipitate-poor plasma [39] thanks to the robustness of the 5%/pH 5.5 caprylic acid step to precipitate non-Ig proteins [19]. Thus, the process does require training and basic equipment and facility, but is more feasible than current fractionation technologies for implementation in low or medium income countries.

The enrichment factor found for the two specific immunoglobulins monitored (anti-hepatitis B and anti-rubella IgG) shows the capacity of the process to concentrate hyperimmune IgG. This shows the feasibility of applying this production concept to the preparation of IgG from convalescent plasma. This purification/viral inactivation process, based on small volume and disposable equipment, could be ideal for the preparation of hyperimmune IgG from convalescent plasma in infectious outbreaks, as seen currently in West African countries exposed to the Ebola virus [6,40].

### Conclusion

Producing a 90% pure immunoglobulin fraction in disposable, single-use devices is feasible. This method could be used to produce immunoglobulins from local plasma in developing countries to protect immunodeficient patients against infectious agents, and could be of interest for preparing hyperimmune IgG from convalescent plasma collected during infectious outbreaks such as the current Ebola virus episode. Clinical evaluations of this preparation in immunodeficient children are on-going and indicate good tolerance and normal IgG half-life.
